# Neoadjuvant immunotherapy for resectable esophageal cancer: A protocol of meta-analysis

**DOI:** 10.1371/journal.pone.0252829

**Published:** 2021-06-04

**Authors:** Guocan Yu, Wenfeng Yu, Xudong Xu, Bo Ye, Liwei Yao

**Affiliations:** 1 Department of Thoracic Surgery, Affiliated Hangzhou Chest Hospital, Zhejiang University School of Medicine, Hangzhou, Zhejiang, China; 2 Department of Nursing, Affiliated Hangzhou Chest Hospital, Zhejiang University School of Medicine, Hangzhou, Zhejiang, China; Zagazig University, EGYPT

## Abstract

**Background:**

Esophageal cancer is a highly malignant cancer with a very poor prognosis. For resectable esophageal cancer, neoadjuvant treatment could improve the prognosis of esophageal cancer. However, current clinical neoadjuvant treatment options for esophageal cancer are still limited. The application of immunotherapy is a potentially beneficial new neoadjuvant treatment option for esophageal cancer. The objective of this meta-analysis is to evaluate the efficacy and safety of immunotherapy for the neoadjuvant treatment of esophageal cancer.

**Methods:**

We will search Wanfang Database, SinoMed, China National Knowledge Infrastructure, Embase, Web of Science, Pubmed, and Cochrane Library for relevant articles published before July, 2021. We will also search the unpublished clinical trials of neoadjuvant immunotherapy in esophageal cancer in preprint website (such as bioRXiv and medRxiv) up to July, 2021. We will perform a meta-analysis to evaluate the efficacy and safety of neoadjuvant immunotherapy for resectable esophageal cancer. Randomized controlled trials (RCTs) will be included in this study. The risk of bias will be evaluated for each included study using the Cochrane Handbook for Systematic Reviews of Interventions. We will use RevMan 5.3 software for statistical analysis of the data.

**Results:**

The results of this study will provide evidence of immunotherapy using as neoadjuvant treatment for esophageal cancer. This meta-analysis will be submitted to a peer-reviewed journal seeking for publication.

**Conclusion:**

The results of this study will provide a reliable basis for clinicians and patients to formulate the best pre-surgical treatment plan for resectable esophageal cancer.

**Systematic review registration:**

INPLASY202120026.

## Introduction

Esophageal cancer is one of the most common malignant tumors [1], and has the seventh highest morbidity rate and the sixth highest mortality rate among all malignancies in the world [2]. Esophageal cancer is a tumor with high degree of malignancy and is prone to invasion and metastasis [3, 4]. Despite multiple therapy options, esophageal cancer remains the main source of cancer-related deaths worldwide [5]. A majority of patients with esophageal cancer are diagnosed at locally advanced stage. The 5-year survival rate for locally advanced stages esophageal cancer is still unsatisfactory [5, 6].

Surgery is still the radical treatment for esophageal cancer [7]. However, for resectable locally advanced esophageal cancer, a direct surgery is sometimes difficult. In such cases, preoperative neoadjuvant therapy is usually used [8]. Several studies have demonstrated that neoadjuvant chemoradiotherapy improved the survival rate of patients with esophageal cancer [9–11]. However, sometimes the treatment-related adverse events (TRAEs) of neoadjuvant chemoradiotherapy are severe and not well tolerated by patients [12]. For this group of patients, there is an urgent need for a new and more effective and safer neoadjuvant therapy.

In recent years, immunotherapy has opened up a whole new field for the treatment of esophageal cancer [13, 14]. Several clinical studies have shown the advantages of immunotherapy over chemotherapy in the treatment of esophageal cancer [15, 16]. However, the role of immunotherapy in the neoadjuvant treatment of esophageal cancer is still lacking in the evidence of evidence-based medicine. For this purpose, we will conduct a meta-analysis to evaluate the efficacy and safety of neoadjuvant immunotherapy in the treatment of resectable esophageal cancer.

## Methods

The preferred reporting items for systematic review and meta-analysis protocols (PRISMA-P) statement will be followed to conducted this protocol [17]. The final results of this research will be reported adhere to the Preferred Reporting Items for Systematic Reviews and Meta-Analyses (PRISMA) statements [18]. This protocol has been registered on INPLASY (International Platform of Registered Systematic Review and Meta-analysis Protocols), the registration number is INPLASY202120026 [19].

### Information sources

We will search Wanfang Database, SinoMed, China National Knowledge Infrastructure (CNKI), Embase, Web of Science, Pubmed, and Cochrane Library for relevant articles that may be eligible for inclusion published before July, 2021. We will also identify other articles from the review’s references that meet the criteria. We will also search the unpublished clinical trials of neoadjuvant immunotherapy in esophageal cancer in preprint website (such as bioRXiv and medRxiv) up to July, 2021.

### Search strategy

The two reviewers (Guocan Yu and Wenfeng Yu) will use the keywords or subject terms corresponding to medical subject heading (MeSH) terms to design search strategies for each database and perform searches in each database to identify eligible articles. No language restrictions will be applied in the search process. The search strategies for PubMed are showed as follows:

#1 "Immunotherapy"[Mesh] OR Immunotherapies OR

#2 "Programmed Cell Death 1 Receptor"[Mesh] OR “PD-1 Protein” OR “PD 1 Protein” OR “PD-1 Receptor” OR “PD 1 Receptor” OR “Receptor, PD-1” OR “Antigens, CD279” OR “CD279 Antigens” OR “CD279 Antigen” OR “Antigen, CD279” OR “PD1 Receptor” OR “Receptor, PD1” OR “Programmed Cell Death Protein 1” OR “Programmed Cell Death 1 Protein”

#3 "Immune Checkpoint Inhibitors"[Mesh] OR “Checkpoint Inhibitors, Immune” OR “Immune Checkpoint Inhibitor” OR “Checkpoint Inhibitor, Immune” OR “Immune Checkpoint Blockers” OR “Checkpoint Blockers, Immune” OR “Immune Checkpoint Blockade” OR “Checkpoint Blockade, Immune” OR “Immune Checkpoint Inhibition” OR “Checkpoint Inhibition, Immune” OR “PD-L1 Inhibitors” OR “PD L1 Inhibitors” OR “PD-L1 Inhibitor” OR “PD L1 Inhibitor” OR “Programmed Death-Ligand 1 Inhibitors” OR “Programmed Death Ligand 1 Inhibitors” OR “CTLA-4 Inhibitors” OR “CTLA 4 Inhibitors” OR “CTLA-4 Inhibitor” OR “CTLA 4 Inhibitor” OR “Cytotoxic T-Lymphocyte-Associated Protein 4 Inhibitors” OR “Cytotoxic T Lymphocyte Associated Protein 4 Inhibitors” OR “Cytotoxic T-Lymphocyte-Associated Protein 4 Inhibitor” OR “Cytotoxic T Lymphocyte Associated Protein 4 Inhibitor” OR “PD-1 Inhibitors” OR “PD 1 Inhibitors” OR “PD-1 Inhibitor” OR “Inhibitor, PD-1” OR “PD 1 Inhibitor” OR “Programmed Cell Death Protein 1 Inhibitor” OR “Programmed Cell Death Protein 1 Inhibitors” OR “PD-1-PD-L1 Blockade” OR “Blockade, PD-1-PD-L1” OR “PD 1 PD L1 Blockade”

#4 #1 OR #2 OR #3

#5 "Esophageal Neoplasms"[Mesh] OR “Esophageal Neoplasm” OR “Neoplasm, Esophageal” OR “Esophagus Neoplasm” OR “Esophagus Neoplasms” OR “Neoplasm, Esophagus” OR “Neoplasms, Esophagus” OR “Neoplasms, Esophageal” OR “Cancer of Esophagus” OR “Cancer of the Esophagus” OR “Esophagus Cancer” OR “Cancer, Esophagus” OR “Cancers, Esophagus” OR “Esophagus Cancers” OR “Esophageal Cancer” OR “Cancer, Esophageal” OR “Cancers, Esophageal” OR “Esophageal Cancers”

#6 "Neoadjuvant Therapy"[Mesh] OR “Neoadjuvant Therapies” OR “Therapy, Neoadjuvant” OR “Neoadjuvant Treatment” OR “Neoadjuvant Treatments” OR “Treatment, Neoadjuvant” OR “Neoadjuvant Radiotherapy” OR “Neoadjuvant Radiotherapies” OR “Radiotherapy, Neoadjuvant” OR “Neoadjuvant Radiation Treatment” OR “Neoadjuvant Radiation Treatments” OR “Radiation Treatment, Neoadjuvant” OR “Treatment, Neoadjuvant Radiation” OR “Neoadjuvant Radiation Therapy” OR “Neoadjuvant Radiation Therapies” OR “Radiation Therapy, Neoadjuvant” OR “Therapy, Neoadjuvant Radiation” OR “Neoadjuvant Radiation” OR “Neoadjuvant Radiations” OR “Radiation, Neoadjuvant” OR “Neoadjuvant Systemic Therapy” OR “Neoadjuvant Systemic Therapies” OR “Systemic Therapy, Neoadjuvant” OR “Therapy, Neoadjuvant Systemic” OR “Neoadjuvant Systemic Treatment” OR “Neoadjuvant Systemic Treatments” OR “Systemic Treatment, Neoadjuvant” OR “Treatment, Neoadjuvant Systemic” OR “Neoadjuvant Chemotherapy” OR “Chemotherapy, Neoadjuvant” OR “Neoadjuvant Chemotherapies” OR “Neoadjuvant Chemotherapy Treatment” OR “Chemotherapy Treatment, Neoadjuvant” OR “Neoadjuvant Chemotherapy Treatments” OR “Treatment, Neoadjuvant Chemotherapy” OR “Neoadjuvant Chemoradiotherapy” OR “Chemoradiotherapy, Neoadjuvant” OR “Neoadjuvant Chemoradiotherapies” OR “Neoadjuvant Chemoradiation Therapy” OR “Chemoradiation Therapy, Neoadjuvant” OR “Neoadjuvant Chemoradiation Therapies” OR “Therapy, Neoadjuvant Chemoradiation” OR “Neoadjuvant Chemoradiation Treatment” OR “Chemoradiation Treatment, Neoadjuvant” OR “Neoadjuvant Chemoradiation Treatments” OR “Treatment, Neoadjuvant Chemoradiation” OR “Neoadjuvant Chemoradiation” OR “Chemoradiation, Neoadjuvant” OR “Neoadjuvant Chemoradiations” OR “pre-surgical” OR “presurgical” OR “pre-operative” OR “preoperative” OR neoadjuvant

#7 #4 AND #5 AND #6

The SinoMed, Wanfang Database, CNKI, Embase, and Cochrane Library search strategies will be similar to Pubmed.

### Eligibility criteria

#### Types of studies

Clinical randomized controlled trials (RCTs), which must have been completed and evaluated the efficacy and safety of neoadjuvant immunotherapy in resectable esophageal cancer. Reviews, repeated publications, articles published not using Chinese or English, studies with less than 10 patients, and case reports will be excluded.

#### Types of participants

Participants with resectable esophageal cancer confirmed by histopathology or cytopathology and immune checkpoint inhibitors (ICIs) were used as neoadjuvant therapy. There will be no restrictions on age, gender, and ethnicity.

#### Types of interventions

Immunotherapy (including all currently known ICIs) alone or immunotherapy plus other therapy as neoadjuvant treatment for resectable esophageal cancer.

#### Comparators

Other treatments (not include immunotherapy) as neoadjuvant therapy for resectable esophageal cancer (not mandatory).

#### Outcomes

Major pathological response (MPR), pathological complete response (pCR), the incidence of TRAE, surgical resection rate, the incidence of surgical complications, and surgical delay rate will be the key clinical outcomes.

### Study selection

Endnote X9.2 software will be used to manage the articles obtained by searching the relevant databases. In the first step, two reviewers (Guocan Yu and Wenfeng Yu) will independently filter duplicate articles through Endnote and exclude them, then exclude articles that do not meet the inclusion criteria by investigating the title and abstract, and finally screen the full text of remaining articles to finalize the eligible articles. If there are disagreements between the two reviewers, a discussion with the third reviewer (Liwei Yao) will be conducted as a way to resolve the disagreements.

### Data extraction

The same two reviewers (Guocan Yu and Wenfeng Yu) as in the study selection phase will independently extract the necessary data from the articles included. Cross-check will be done to find controversial data and resolve by discussing with a third author (Liwei Yao).

The following data from the included articles will be extracted: first author name, year of publication, countries, article type, clinical trial, registration number, study phase, masking, main inclusion criteria, type of pathology, the ICI drug, ICIs dose, programmed cell death-ligand-1 [PD-L1]/ programmed cell death 1 [PD-1] expression levels in tumoral and immune cells, expected inclusion, size of sample, male, median age, MPR, pCR, incidence of TRAE, surgical resection rate, incidence of surgical complication, and surgical delay rate.

### Risk of bias

Two authors (Guocan Yu and Wenfeng Yu) will independently assess the risk of bias of each article included. The Cochrane Handbook for Systematic Reviews of Interventions will be used for the evaluation of the risk of bias [20]. We will assess the risk of bias according to the following ranges: selection bias, performance bias, detection bias, attrition bias, reporting bias, and other biases. Each domain will be assessed as high, low or uncertain risk of bias. The risk of bias graph will be reported to demonstrate the results and details of assessment.

### Statistical analysis

We will use Review Manager software, version 5.3 (Copenhagen: The Nordic Cochrane Centre, The Cochrane Collaboration, 2014) for statistical analysis of the meta-analysis. We will calculate pooled risk ratios (HRs) for MPR, pCR, incidence of TRAE, surgical resection rate, incidence of surgical complication, and surgical delay rate with comparative binary data in RevMan 5.3. HR with 95% confidence interval (CI) will be the effect measures. The statistical heterogeneity between studies will be evaluated by the Q-statistic [21]. The *P*-value of the Q-statistic < 0.1 or an I^2^ > 50% will be considered as statistically significant heterogeneity between studies [22]. Data will be analyzed using a fixed-effects model if the heterogeneity is insignificant and a random-effects model if the heterogeneity is significant [23]. A *P* < 0.05 was considered statistically different.

#### Subgroup analysis

When significant heterogeneity exists and sufficient data are available, we will conduct subgroup analysis to further explore the sources of heterogeneity. We will conduct subgroup analysis of each parameter (such as article type, masking, type of pathology, the ICI drug, PD-L1/PD-1 expression levels, sex, age), when the extracted data are sufficient.

#### Sensitivity analysis

Sensitivity analysis will be conducted to evaluate the reliability and robustness of the aggregation results via eliminating studies with high bias risk.

### Publication bias

We will use funnel plots and Egger test to assess publication bias when more than 10 eligible articles are included [24]. If publication bias is suspected in a study, we will consult the corresponding author for more information. If publication bias does exist, we will use the fill and trim method to further analyze publication bias in the studies.

### Evidence evaluation

We will evaluate all the strength of the body of evidence according to The Grading of Recommendations Assessment, Development and Evaluation (GRADE) guideline [25]. The quality of evidence will be classified into 4 levels: high, moderate, low, and very low.

## Discussion

Esophageal cancer is a highly malignant cancer with a very poor prognosis. For resectable locally advanced esophageal cancer, neoadjuvant treatment could improve the prognosis of esophageal cancer. Effective neoadjuvant treatment options are needed, but current clinical treatment options are still limited for esophageal cancer. The application of immunotherapy is a potentially beneficial new neoadjuvant treatment option for esophageal cancer. To our best knowledge, this will be the first meta-analysis to evaluate the efficacy and safety of immunotherapy for the neoadjuvant treatment of esophageal cancer. The results of this study will provide a reliable basis for clinicians and patients to formulate the best pre-surgical treatment plan for resectable esophageal cancer.

## Supporting information

S1 Checklist Preferred reporting items for systematic review and meta-analysis protocols. (DOC)
